# Clinical manifestation for immunoglobulin A deficiency: a systematic review and meta-analysis

**DOI:** 10.1186/s13223-023-00826-y

**Published:** 2023-08-28

**Authors:** Ahmad Vosughimotlagh, Seyed Erfan Rasouli, Hosein Rafiemanesh, Molood Safarirad, Niusha Sharifinejad, Atossa Madanipour, Maria Marluce Dos Santos Vilela, Edyta Heropolitańska-Pliszka, Gholamreza Azizi

**Affiliations:** 1 Department of Pediatrics, North Khorasan University of Medical Sciences, Bojnurd, Iran; 2 Non-communicable Diseases Research Center, Alborz University of Medical Sciences, Karaj, Iran; 3 Student Research Committee, Alborz University of Medical Sciences, Karaj, Iran; 4 Center for Investigation in Pediatrics, Pediatrics Department, Faculty of Medical Sciences, State University of Campinas (UNICAMP), Campinas, SP Brazil; 5 Department of Immunology, Children’s Memorial Health Institute, Warsaw, Poland; 6 Department of Neurology, Thomas Jefferson University, Philadelphia, PA USA

**Keywords:** Immunoglobulin A deficiency, Autoimmune disorders, Infections, Malignancy, Allergic diseases

## Abstract

**Objectives:**

Immunoglobulin A deficiency (IgAD) is a common disease with an unknown genetic defect, characterized by the decreased or absent IgA with other isotypes normal, normal subclasses, and specific antibodies. Patients with this disorder represent a spectrum of clinical manifestations including infections, autoimmune disorders, malignancy, and allergic diseases. The current study aimed to evaluate their prevalence and categorized them.

**Methods:**

We searched PubMed, Web of Science, and Scopus databases to find eligible studies from the earliest available date to January 2022 with standard keywords. Pooled estimates of clinical manifestations prevalence and the corresponding 95% confidence intervals were calculated using random-effects models.

**Results:**

The most prevalent clinical manifestations belonged to infection (64.8%) followed by allergic diseases (26.16%) and autoimmunity (22.0%), respectively. In selective IgA deficiency patients as the largest group of IgAD in current study, celiac disease (6.57%), Inflammatory bowel disease (4.01%), and rheumatoid arthritis (3.80%) were the most prevalent autoimmunity. Meanwhile, the most frequent infection was respiratory tract infection, fungal infection, and gastrointestinal infection at 50.74%, 18.48%, and 15.79%, respectively. In addition, the pooled prevalence of asthma, allergic rhinitis, and allergic conjunctivitis were 19.06%, 15.46%, and 11.68%, respectively which were reported as the most widespread allergic diseases.

**Conclusions:**

Our results showed that apart from undiagnosed IgAD patients, IgAD patients represent a wide range of clinical manifestations. Infection, allergy, and autoimmunity are the most common clinical manifestations. The concurrent presence of IgA and IgG subtypes deficiency could be associated with increased susceptibility to infection. Considering the probability of developing new clinical complications during follow-up, periodic assessments of IgAD patients should be inspected.

**Supplementary Information:**

The online version contains supplementary material available at 10.1186/s13223-023-00826-y.

## Introduction

Selective immunoglobulin A deficiency (SIgAD) is defined as the decreased or absent level of serum immunoglobulin A (IgA) in the presence of normal serum levels of immunoglobulin G (IgG) and immunoglobulin M (IgM) in a patient older than 4 years of age, in whom other causes of hypogammaglobulinemia have been excluded [1]. In general, serum IgA level of less than 7 mg/dL (0.07 g/L) is considered SIgAD [1] which consider the largest group of IgAD. Meanwhile, when serum IgA level is higher than 7 mg/dL but two standard deviations below normal for age, the condition may be referred to as partial immunoglobulin A deficiency (PIgAD) [1]. In addition, some of those with IgA deficiency have been found to also have IgG subclass deficiency [2].

SIgAD is the most common humoral immunodeficiency [3] with an estimated occurrence from about 1:3000 to even 1:150, depending on the population [4]. The spectrum of SIgAD clinical manifestations is varied. SIgAD is distinguished from other immunodeficiencies as more than 50% of the affected individuals do not show any clinical symptoms [5] which is identified by coincidental findings [6] in laboratory screening of normal individuals or among blood donation. Meanwhile, the rest of the cases have manifestations such as recurrent infections [7, 8], allergies [1, 8], autoimmune diseases [9, 10], and an increased risk of cancer [11].

Altogether, the clinical symptoms of immunodeficiency and immune dysregulation are much higher in SIgAD than in the normal population [6]. The most common disease presentation in symptomatic patients is recurrent infections, especially respiratory infections [12]. Autoimmunity with a prevalence of 25–31% is another clinical complication of patients with SIgAD [13, 14]. In addition, between 18% and 56% of SIgAD patients suffer from one or more allergies [13]. Because of the variability of clinical manifestations, symptomatic patients visit various physicians of different specialties in search of a diagnosis, which increases the risk of missing the overarching clinical pattern and thereby overlooking the underlying SIgAD.

In this study, to improve our insight into the early presentation of SIgAD and to assist physicians in its timely detection, we sought to systematically evaluate the prevalence of various types of clinical manifestations in patients with three types of IgAD. The results could assist primary care physicians in diagnosing IgAD and provide an overview of patients’ clinical manifestations leading to early detection followed by appropriate preventive interventions.

## Materials and methods

This systematic review and meta-analysis study has been conducted following Cochrane Handbook and reported using Systematic Reviews and reported using Preferred Reporting Items for Systematic Reviews and Meta-Analyses (PRISMA) checklist.

### Search strategy

Our search strategy has three components: (1) first, we conducted an extensive search on international digital databases for published articles, (2) seconds, the hand-searching of the reference section of the review articles was done; and (3) third, Communicating with the experts in the field of primary immunodeficiency diseases (PIDs) and also, reviewing relevant journals for recently published articles by the end of the year 2021. The search was limited to the English language and human studies.

### Databases searched

A systematic literature search was performed using the Web of Science, PubMed, and Scopus databases to collect English documents published by February 2022. Search approach terms were categorized into three groups: (1) “selective IgA deficiency”, “selective IgAD”, “selective IgA def”, “Selective immunoglobulin A def”, “Selective immunoglobulin A deficiency”, “SIgAD”, “SIgA def”, “SIgA deficiency”. (2) “IgAD”, “IgA def”, “IgA deficiency”, “immunoglobulin A def”, “immunoglobulin A deficiency”. (3) “Partial IgAD”, “Partial IgA def”, “Partial IgA deficiency”, “Partial immunoglobulin A def”, “Partial immunoglobulin A deficiency”, “PIgAD”, “PIgA def”, “PIgA deficiency”. The search was conducted using these terms in the titles and abstracts.

### Screening process

The selection process of the obtained documents was performed in two steps: (1) the preliminary screening of all relevant studies based on the titles and abstracts; (2) detailed assessments of available full-texts for eligibility criteria. In addition, references from original articles were further scanned manually for additional studies of interest. The studies qualified for the inclusion criteria: (1) their evaluation subject was the epidemiological, demographical, and clinical features of patients with a definite diagnosis of SIgAD so that reported their main or alternative outcome of interest as clinical presentation incidence or prevalence. (2) The articles were written in the English language, [3] conducted on human subjects with SIgAD and PIgAD diagnosis and also SIgAD with subclass IgG deficiency. Based on the PAGID (Pan-American Group for Immunodeficiency) or ESID (European Society for Immunodeficiencies) diagnostic criteria. In addition, due to the high prevalence of pneumonia and sinusitis, these two variables were analyzed separately from other respiratory tract infections. Furthermore, for studies with overlapping data, those with larger samples and more detailed information were included. Both steps of study selection were taken independently by two reviewers and any disagreement was resolved via consensus with a third reviewer.

### Data extraction

Data were extracted from all included studies into a standardized Microsoft Excel spreadsheet. The following data were collected: name of the first author, published year, the original country of the study, study design, and the study population characteristics including demographic, clinical, and epidemiological data. Patients were divided into three groups: [1] patients with serum IgA level of less than 7 mg/dL (0.07 g/L) (SIgAD), [2] patients with serum IgA level higher than 7 mg/dL but two standard deviations below normal for age (PIgAD), and [3] IgAD patients with co-occurrence of IgG subclass deficiency. In addition, we contacted the corresponding authors to evaluate unavailable data.

### Statistical analysis

All the eligible studies were included in the synthesis after a systematic review. The meta-analysis was performed using the random-effects model to assess the pooled prevalence of the main clinical findings in SIgAD patients. A graphical display of the reported clinical findings prevalence in the included studies was presented in the forest plot. The heterogeneity was tested using the I2 square statistic, which describes the percentage of variation between studies that is due to heterogeneity rather than chance. The meta-analysis was performed by Stata v.14 (StataCorp, College Station, TX).

## Results

### Study selection

**Fig. 1:**
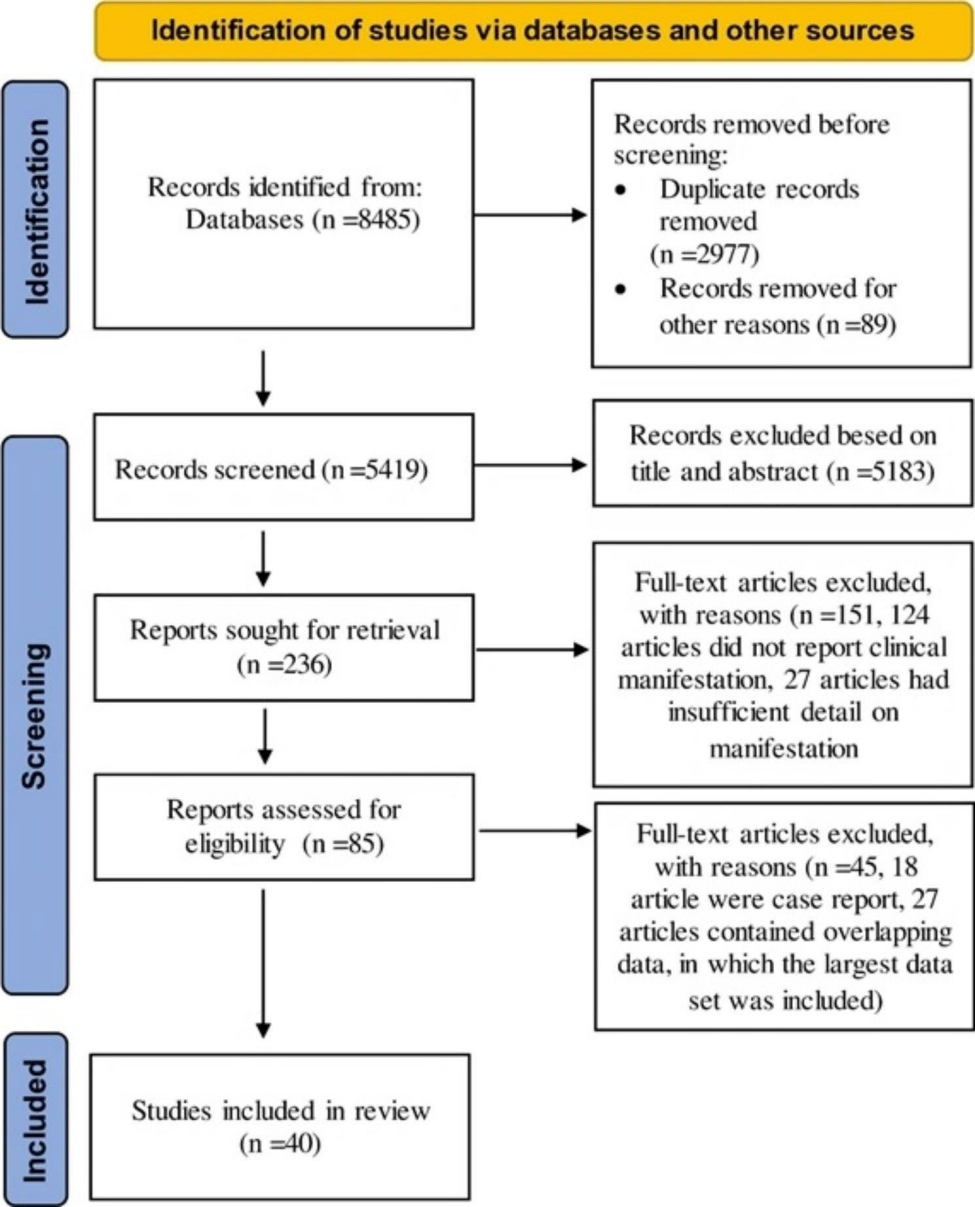
Flow chart of selected studies

The literature search results and selection process based on the PRISMA flow chart for systematic reviews are shown in Fig. 1. A total of 8485 articles were retrieved from the initial search in different databases, from which 2977 were duplicated studies. After the screening of 5419 studies for titles and abstracts, 236 articles were selected and the full texts were assessed. 151 articles were excluded as they did not present data on clinical manifestation (Fig. 1). Furthermore, 18 case report articles were excluded and 27 articles excluded that contained overlapping data, in which the largest data set was included.

### Study characteristics

The final extracted articles were 40 but according to clinical manifestations, there was an overlap between studies, and some articles reported more than one type of IgAD. So, in terms of IgAD types, the final extracted articles include 37, 6, and 3 papers for SIgAD, PIgAD, and IgAD patients with IgG subclass deficiency, respectively. The studies have been conducted in 39 unique countries [mostly originated in Finland (n = 4), and Turkey, Italy, and Israel (each of them, n = 3)], the oldest study was published in 1971, and the latest in 2021. The sample sizes of patients with SIgAD, PIgAD, and IgAD patients with IgG subclass deficiency in included articles varied from 4 patients with IgA deficient patients with IgG subclass deficiency of the Affiliated University Children’s Hospital in Berne up to 2100 SIgAD patients in a study through six university hospitals in Sweden. The characteristics of the included studies in this systematic review are depicted in Table S1.

### The frequency of clinical manifestations in IgAD

The pooled prevalence of infection (in 1,056 IgAD patients), autoimmunity (in 1,660 IgAD patients), at least one allergic disease (in 1,429 IgAD patients), and malignancy (in 904 IgAD patients) was 64.0% (95% CI: 49.8, 77.2%; I2 = 95.4%), 21.9% (95% CI: 17.9, 26.1%; I2 = 71.6%), 29.0% (95% CI: 20.5, 38.2%; I2 = 92.3%), and 3.5% (95% CI: 1.0, 7.1%; I2 = 75.5%), respectively. In this regard, celiac disease [6.57% (95%CI: 3.83 to 9.88)], respiratory tract infection [50.74% (95%CI: 40.24 to 61.21)], and asthma [19.06% (95%CI: 11.91 to 27.34)] were the most prevalence presentation in the group of autoimmunity, infection, and allergic diseases, respectively.

#### Infection prevalence in SIgAD

**Fig. 2:**
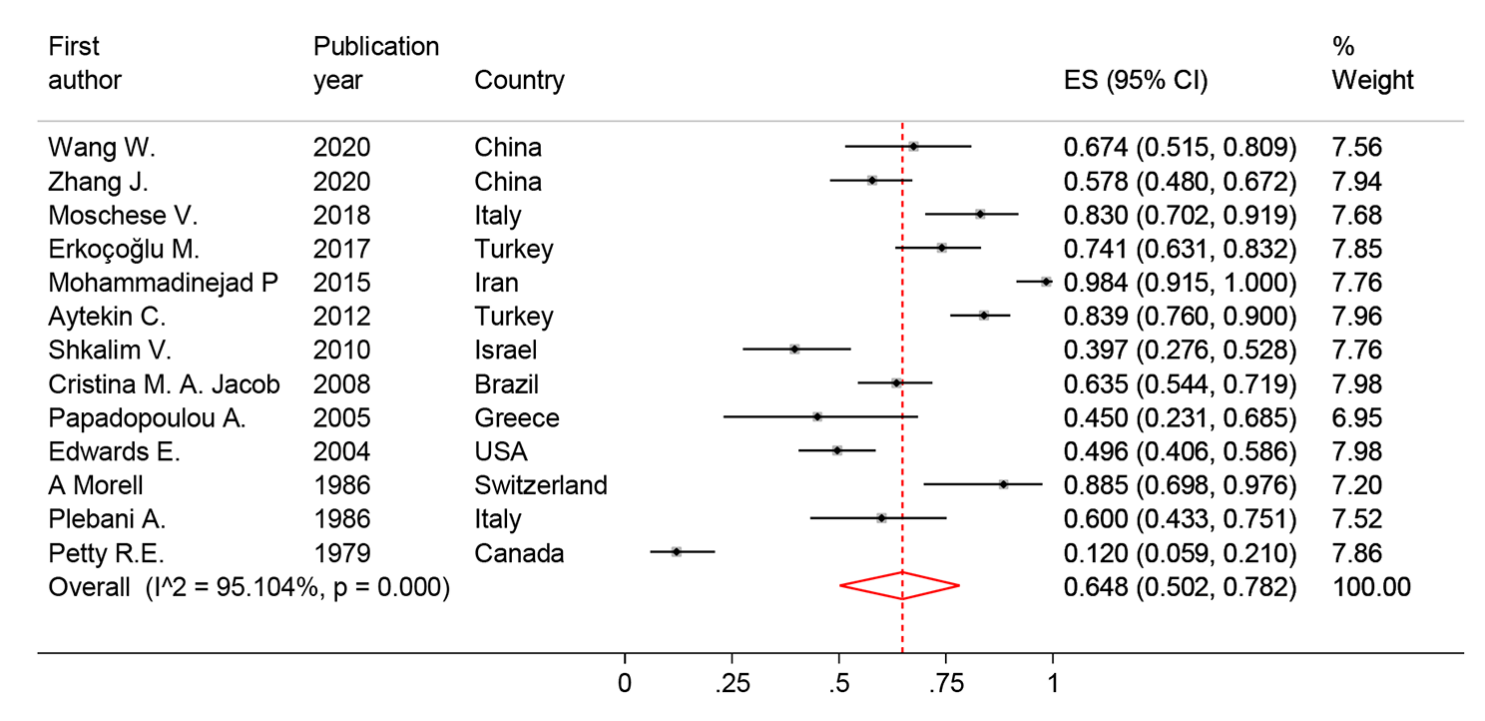
The forest plot and pooled prevalence of the infection in SIgAD

The prevalence of at least one infection was reported in 13 distinct studies and varied from 12.04% by Petty R.E, et al. from Canada (95% CI: 0.059–0.210) [15] to 98.41% by Mohammadinejad P, et al., from Iran (95% CI: 0.915-0.1000) [16]. The pooled prevalence of infection in the 952 study population was 64.8% (95% CI: 50.2, 78.2%; I2 = 95.1%) **(**Fig. 2**)**. Eleven types of infectious presentation were reported by the current study investigations. Based on the meta-analysis result, respiratory tract infection (50.74%), fungal infection (18.48%), and Gastrointestinal infection (15.79%) were the most frequent infection in SIgAD patients **(**Table 1**)**.

**Table 1:** The pooled prevalence of infections in selective IgA deficiency patients

Type of infection	Ref	N*, sample size	ES% (95%CI); I2%
Respiratory tract infection	(16–19, 22–24, 27, 29, 32, 33, 45, 51, 67–71)	19, 1389	50.74 (40.24 to 61.21); 93.08
Pneumonia**	(8, 18, 19, 21, 25, 27, 29, 45, 67–69, 72, 73)	13, 892	12.15 (5.00 to 21.53); 92.07
Otitis	(8, 19, 21, 25, 29, 72)	6, 416	7.19 (2.61 to 13.51); 74.95
Sinusitis**	(8, 19, 29, 67)	4; 294	8.13 (0.54 to 21.64); 88.99
Gastrointestinal infection	(16, 22, 23, 27, 29, 32)	6, 352	15.79 (6.52 to 27.83); 85.54
Skin infection	(8, 15, 16, 19, 32, 45)	6, 456	15.31 (2.17 to 36.27); 96.15
Conjunctivitis	(8, 21)	2, 77	12.86 (6.01 to 21.55); NA
Bacterial infection	(8, 33)	2, 150	8.73 (4.52 to 14.00); NA
Viral infection	(15, 23–25, 32, 51, 67)	7, 507	3.99 (0.69 to 9.21); 79.63
Parasitic infection	(8, 23)	2, 85	1.72 (0.0 to 6.25); NA
Fungal infection	(8, 23)	2, 85	18.48 (10.72 to 27.65); NA

*Number of studies in Meta-analysis

** Due to the high prevalence of pneumonia and sinusitis, these two variables were analyzed separately from other respiratory tract infections

#### Autoimmunity prevalence in SIgAD

**Fig. 3:**
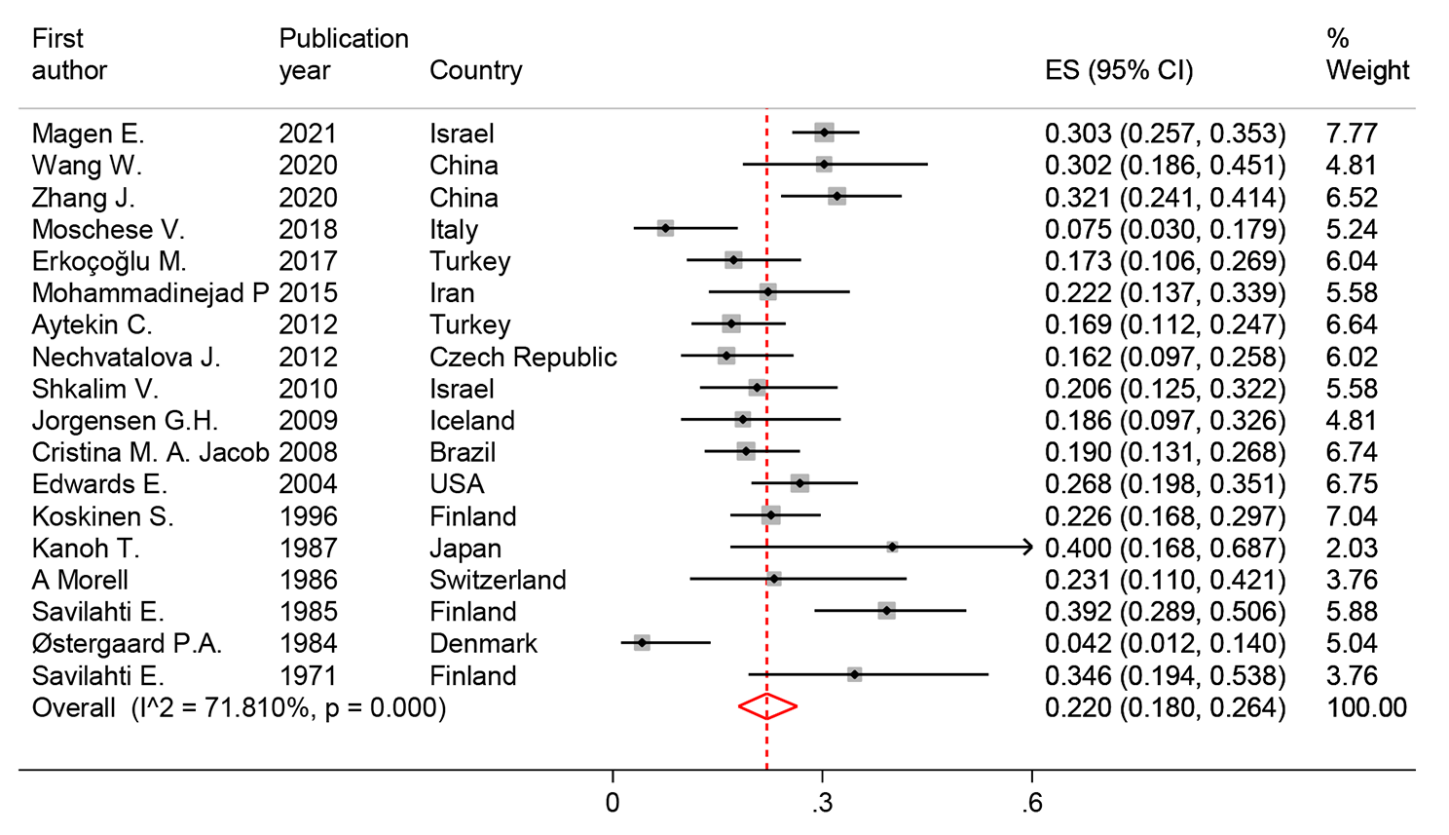
The forest plot and pooled prevalence of autoimmunity in SIgAD

The prevalence of at least one autoimmunity in SIgAD patients was assessed in 18 studies and ranged from 4.2% by Østergaard P.A. from Denmark (95% CI: 0.012–0.140) [17] to 39.2% by Kanoh T., et al. from Japan (95% CI: 0.168–0.687) [18]. The pooled prevalence of autoimmunity in the 1,596 study populations was 22.0% (95% CI: 18.0, 26.4%; I2 = 71.8%) **(**Fig. 3**)**. Eighteen types of autoimmune manifestations were reported by the current study investigations. Based on meta-analysis results, celiac disease (6.57%), inflammatory bowel disease (IBD) (4.01%), and rheumatoid arthritis (3.80%) were the most prevalent autoimmunity in SIgAD patients, respectively **(**Table 2**)**.

**Table 2:** The pooled prevalence of autoimmunity in selective IgA deficiency patients

Type of autoimmunity	Ref	N*, sample size	ES% (95%CI); I2%
AIHA	(16, 18, 29, 45)	4, 326	1.48 (0.0 to 4.68); 44.75
ITP	(16, 18, 19, 22, 29, 31, 33, 74)	8, 577	1.61 (0.41 to 3.34); 20.33
Evans syndrome	(18, 19)	2, 119	0.42 (0.0 to 3.54); NA
Autoimmune thyroiditis	(15, 18, 22, 23, 31, 32, 45, 51, 53, 67, 70, 75)	12, 2868	2.99 (1.52 to 4.81); 51.21
IBD	(18, 25, 51, 53, 70, 73)	6, 2429	4.01 (0.71 to 9.11); 84.96
IDDM	(18, 23, 25, 29, 31, 33, 53, 67, 70, 73, 75)	11, 2801	3.29 (2.02 to 4.79); 28.21
Celiac disease	(8, 18, 23, 25, 31, 33, 40, 45, 51, 53, 68, 70–73, 76, 77)	17, 3813	6.57 (3.83 to 9.88); 86.91
Vitiligo	(18, 25, 31, 40, 45, 51, 75)	7, 872	1.40 (0.62 to 2.19); 0.0
Rheumatoid arthritis	(15, 16, 18, 19, 22, 29, 31, 33, 45, 51, 53, 67, 69–72, 74)	17, 3225	3.80 (1.66 to 6.56); 81.49
SLE	(15, 18, 19, 22, 29, 32, 40, 45, 53, 70, 74)	11, 3088	3.56 (1.07 to 7.09); 87.95
Sjogren syndrome	(18, 19, 40, 51, 67, 70, 74)	7, 768	0.94 (0.08 to 2.38); 25.67
Myasthenia gravis	(18, 53)	2, 2110	< 0.001 (0.0 to < 0.001); NA
Psoriasis	(16, 18, 19, 25, 33, 40, 51, 69, 74)	9, 939	1.47 (0.64 to 2.54); 0.0
Pernicious anemia	(18, 25, 29, 51, 74)	5, 402	0.70 (0.0 to 2.16); 0.0

* Number of studies in Meta-analysis

AIHA: Autoimmune hemolytic anemia, ITP: Idiopathic thrombocytopenic purpura, GBS: Guillain-Barré syndrome,

IBD: Inflammatory bowel disease, IDDM: Insulin-dependent diabetes mellitus, SLE: systemic lupus erythematosus

#### Allergic diseases prevalence in SIgAD

**Fig. 4:**
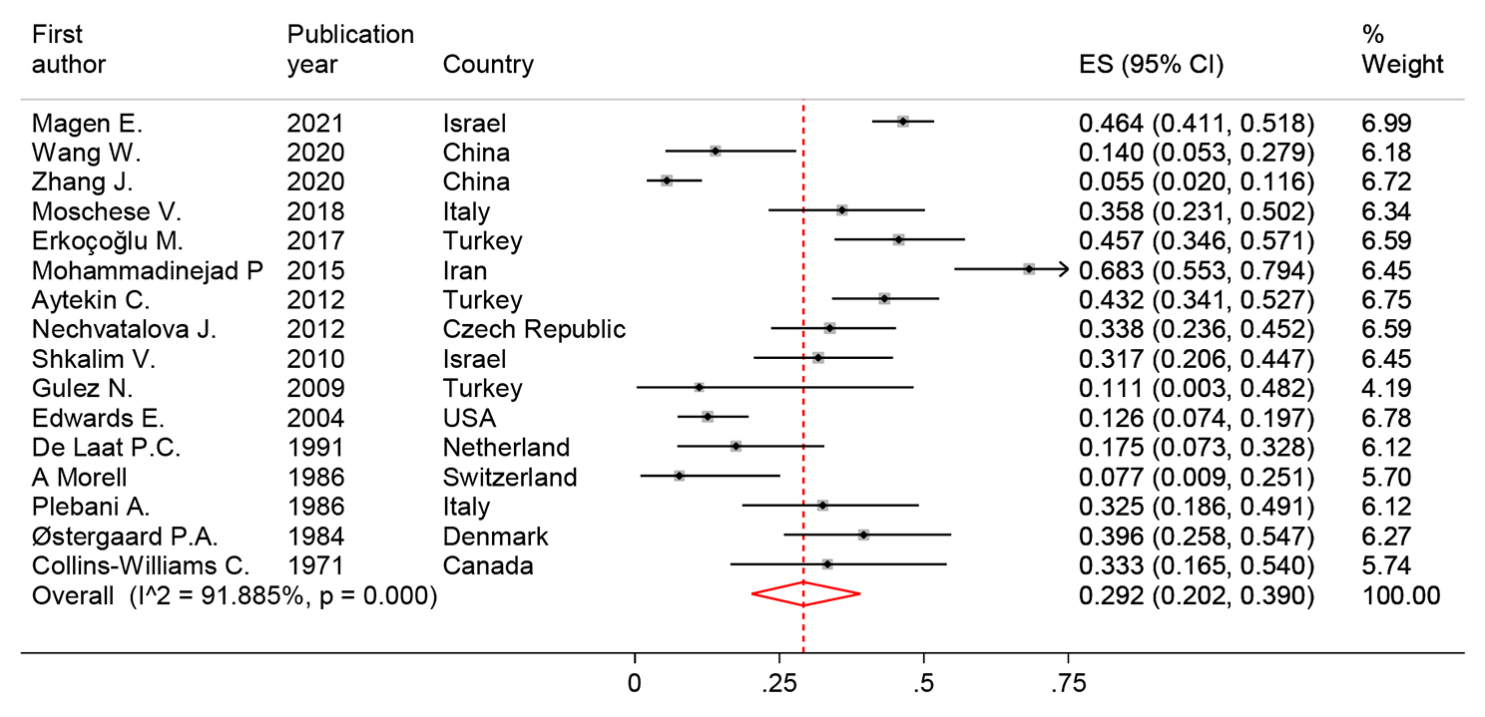
The forest plot and pooled prevalence of Allergic diseases in SIgAD

The prevalence of at least one allergic disease was determined in 16 different studies and fluctuated from 5.50% by Junwu Zhang, et al. from China (95% CI: 0.020–0.116) [19] to 68.25% by Mohammadinejad P, et al., from Iran (95% CI: 0.553–0.794) [16]. The pooled prevalence of at least one allergic disease in the 1,274 study population was 29.16% (95% CI: 20.17, 39.01%; I2 = 91.9%) **(**Fig. 4**)**. Eight types of allergic diseases were reported by the current study investigations. Based on meta-analysis results, the pooled prevalence of asthma, allergic rhinitis, and allergic conjunctivitis were 19.06%, 15.46%, and 11.68%, respectively which were reported as the most widespread allergic diseases in SIgAD patients **(**Table 3**)**.

**Table 3:** The pooled prevalence of allergic diseases in selective IgA deficiency patients

	Ref	N*, sample size	ES% (95%CI); I2%
Asthma	(8, 17, 19, 21, 23, 25, 27, 30–33, 40, 45, 67, 69, 70, 72, 73)	18, 1355	19.06 (11.91 to 27.34); 91.58
Allergic rhinitis	(8, 17, 21, 23, 25, 31–33, 40, 51, 69, 71, 72)	13, 1069	15.49 (9.90 to 21.98); 83.60
Allergic conjunctivitis	(8, 23, 32, 51)	4, 287	11.68 (2.08 to 26.67); 88.50
Allergic bronchopulmonary Aspergillosis	(32, 68)	2, 227	3.68 (1.44 to 6.72); NA
Food allergy	(8, 31–33, 40, 45, 51, 70)	8, 980	1.79 (0.47 to 3.71); 60.35
Eczema	(8, 17, 21, 23, 25, 27, 30–33, 40, 45, 51, 69, 72)	15, 1242	8.27 (5.24 to 11.83); 71.41
Urticaria	(8, 19, 25, 32, 33, 69)	6, 392	2.96 (0.70 to 6.30); 49.57

*.Number of studies in Meta-analysis

#### Malignancy and bronchiectasis prevalence in SIgAD

**Fig. 5:**
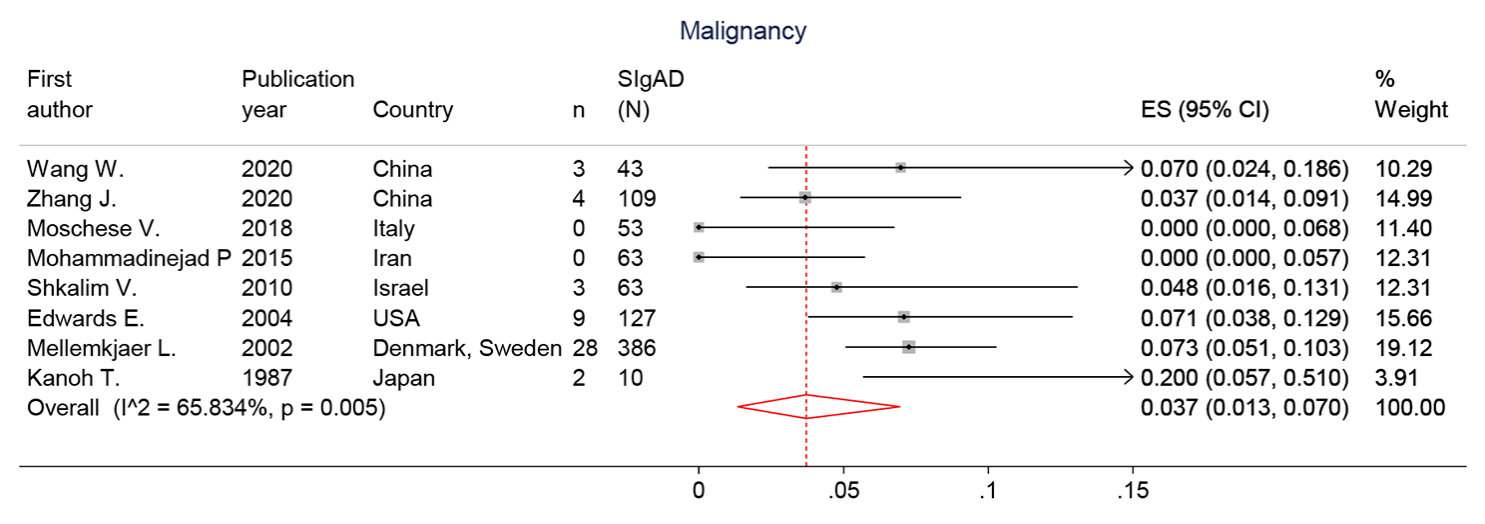
The forest plot and pooled prevalence of the malignancy in SIgAD

The prevalence of malignancy was reported in 8 studies and ranged from 0.0 to 7.3% by Mellemkjaer, et al. from Denmark and Sweden (95% CI: 0.051–0.103) [20]. The pooled prevalence of malignancy in the 854 study population was 3.7% (95% CI: 1.3, 7.0%; I2 = 65.8%) (Fig. 5). Meanwhile, the bronchiectasis frequency was determined in 8 studies and varied from 1.58% by Mohammadinejad P, et al., from Iran (95% CI: 0.003–0.085) [16] to 77.8% by Jelena Živković, et al. from Croatia [21]. The pooled prevalence of bronchiectasis in the 491 study population was 14.9% (95% CI: 2.6, 34.0%; I2 = 95.7%) (Figure S1). The frequency of other clinical manifestations that are not included in the above categories is shown in **Table S2**.

### The clinical manifestations in patients with partial IgAD

The total prevalence of infection in PIgAD patients was determined in three studies. The pooled prevalence of the infection based on this study was 66.36% (95%CI: 34.34 to 92.18). Based on the meta-analysis result, respiratory tract infection was the most frequent infectious manifestation (59.10%), followed by gastrointestinal infection (13.92%) and pneumonia (10.0%) **(Table S3)**.

The prevalence of autoimmunity in PIgAD patients was assessed in two studies which reported zero out of 10 [22] and 9 out of 50 patients [23]. The pooled prevalence of autoimmunity based on this study was 12.87% (95%CI: 4.83 to 23.34). Celiac disease was the most prevalent autoimmunity manifestation (6.66%) followed by autoimmune thyroiditis (2.09%) and rheumatoid arthritis (2.09%) **(Table S3)**.

The prevalence of the allergic diseases was determined in 4 studies with a pooled prevalence of 24.41% (95%CI: 12.50 to 38.50). Allergic rhinitis was the most prevalent allergic disease by 46.55% followed by asthma (27.16%) and eczema (9.96%) **(Table S3).** Also, the incidence of malignancy was assessed in one study for which no cases were reported **(Table S3)**.

### The clinical manifestations in IgA deficient patients with IgG subclass deficiency

The total prevalence of infection in this group was determined in two studies. The pooled prevalence of the infection was 66.36% (95%CI: 34.34 to 92.18). Respiratory tract infection was the most frequent infectious manifestation (87.76%) **(Table S4)**. The prevalence of autoimmunity in IgA deficient patients with IgG subclass deficiency was reported only in one study [2] which reported 17.65% **(Table S4)**. The prevalence of allergic diseases was determined in one study with a pooled prevalence of 17.6% (95%CI: 9.2 to 41.0). Asthma was the only manifestation of allergic diseases which was reported by 13.6% **(Table S4)**. Also, malignancy has been investigated in only one study with 0.0% prevalence **(Table S4)**. Meanwhile, the prevalence of bronchiectasis and chronic diarrhea were illustrated in one study with a pooled prevalence of 4.55% (95%CI: 0.81 to 28.80) and 11.76% (95%CI: 3.29 to 34.34), respectively. It is worth mentioning that, in the extracted studies, some clinical manifestations have been reported for all 4 types of IgG subclass deficiency and due to the limited data in mentioned factors, different types of subclasses can not be reported separately.

## Discussion

IgAD is a primary immunodeficiency described in patients older than 4 years, after excluding other causes of hypogammaglobulinemia and T cell defects [1]. In this study, we provided a comprehensive analysis of the clinical manifestations in IgAD patients and its three main subtypes: SIgAD, PIgAD, and the coexistence of IgAD and IgG subclass deficiency.

Although more than half of IgAD patients remain undiagnosed in their life [5], individuals with SIgAD, as the largest group of both IgAD and predominantly antibody deficiency, represented a wide range of clinical manifestations with various prevalence in different study populations [7]. Our analysis revealed infection to be the most common complication in SIgAD patients (64.8%) with respiratory (50.7%) and GI (15.7%) tract as the main target systems, which is in agreement with previous publications [1, 23–27]. Interestingly, despite former studies insisting on encapsulated bacteria such as *Streptococcus pneumonia* and *Haemophilus influenza* [26], we detected fungal pathogens as the major invading agents (18.5%) highlighting the role of secretory IgA in mucosal immunity against fungi [28]; thus, it could be assumed that fungi are underacknowledged pathogens in SIgAD and patients with a definite diagnosis might benefit from fungal screening. Additionally, the rate of infection considerably varied in different studies; some reported lower [19, 25, 29–31] or higher [16, 23, 32, 33] prevalence of infection than our pooled data. These controversial results could stem from the fact that many individuals may only represent mild infections and therefore not be screened for immunodeficiency. Plus, the lack of diagnostic facilities in developing countries might lead to improper treatment and eventually raise the prevalence of clinical presentations in IgAD.

The other major complications in SIgAD, in the order of prevalence, are allergy (29.16%), autoimmunity (22%), and malignancy (3.7%). IgAD is a heterogenous disease with maltitude pathogenic approaches; the major underlying cause of IgAD centers around B-cell proliferation, maturation, and ultimately immunoglobulin production [34]. Lack of IgA production tend to incite compensatory increase in secretory IgM [35]. Many studies have pointed out an increased risk of atopic diseases in IgAD including bronchial asthma, allergic rhinitis, and atopic dermatitis [25, 31, 36–39]. Although we found allergic diseases far less prevalent than in former reports [16, 23, 31, 40], it ranked second in the clinical manifestations of patients. Asthma was the most prevalent atopy in our analysis of SIgAD cases. However, the association between asthma and SIgAD seems to be conflicting. Although asthma outbreak has been commonly reported in SIgAD patients [41], no significant difference was observed in the incidence of asthma among SIgAD patients and the control group [8]. It is thought that the impaired mucosal immunity in the absence of secretory IgA is mainly responsible for the sensitization against aeroallergens and food allergens in the respiratory and GI tracts [38, 42]. However, they still lack a clear cause-and-effect relationship; arguing whether IgAD promoted allergic reactions or the allergic reactions weakened the mucosal membranes and consequently generated a secondary IgAD [4]. Besides, the influence of nutrition, geographic locations, and genetics should also be taken into consideration, which might explain the low rate of allergy in Chinese patients with SIgAD [19, 32]. Importantly, patients with concomitant IgAD and allergic disease are inclined to develop severe manifestations, especially respiratory tract infections [43].

An association between IgAD and a higher prevalence of autoimmune diseases has been detected [10, 44]. We reported autoimmune diseases in 22% of our SIgAD patients which is substantially greater than the reported proportions from Spain (11.5%), Brazil (19%), Turkey (17.3%), and Israel (20.6%) [13, 25, 31, 45, 46]. This should be noted that these results are mainly from symptomatic cases, as undiagnosed individuals are less likely to be tested for autoimmune diseases or other disorders. Celiac disease (6.57%), inflammatory bowel disease (IBD) (4.01%), and rheumatoid arthritis (3.80%) were the most prevalent autoimmunity in SIgAD patients which is much lower than their prevalence in general population [1.4%, 0.32%, and 0.5-1.0%, respectively] [47–49].

Similar to the prevailing data [10, 14, 25, 31, 33, 36, 45, 50–54], celiac disease was the most frequent autoimmunity in our study. Celiac disease shares a Igfic significance with IgA deficiency possibly through certain HLA haplotypes [55]. Incidentally, due to the paucity of IgA antibodies in SIgAD, celiac disease could be misdiagnosed in the context of this immunodeficiency [56, 57]. Only 9.3% of SIgAD cases with biopsy-confirmed celiac disease had IgG antibodies against TTG, implying physicians need to rely on tissue biopsies in this population [14]. Of note, patients with concurrent celiac disease and IgAD are more likely to have an additional autoimmune disease than individuals with celiac disease alone (67% vs. 23.5%) as well as a lower frequency of GI symptoms (17.7% vs. 66.7%) [58].

PIgAD and the coexistence of IgA and IgG subclass deficiency shared close results in terms of clinical manifestations with SIgAD. Nonetheless, they both had a lower incidence of autoimmunity and allergic diseases without any report of malignancy. However, neither the disease severity nor the occurrence of infections and autoimmune diseases was stated to correlate with serum IgA level [59]. Despite some discrepancy [60], the frequency of severe infections tended to be greater in IgAD with concurrent IgG2 or IgG4 subclass deficiency and/or limited pneumococcal polysaccharide antibody response [29, 61]. Likewise, these patients had a higher rate of infection in the pooled analysis compared to the other groups. Currently, the same size of the PIgAD and concurrent IgA and IgG subclass deficiency prevents a precise conclusion.

In this regard, the pooled prevalence of infection, autoimmunity, and allergic disease in SIgAD were comparable to PIgAD patients. Respiratory tract infection was the most prevalent infection in both groups whereas, gastrointestinal and fungal infection infection had the second position in the PIgAD and SIgAD group. Similar results were also observed in the prevalence of autoimmune manifestations. Generally, the current study indicates that the spectrum of complications in patients with PIgAD and SIgAD has considerable similarities with minor differences in some presentations.

Noteworthily, common variable immunodeficiency (CVID) and IgAD have comparable clinical and immunological features and even IgAD can be seen early in the development of CVID [62]. Progression of IgAD to CVID is a rare phenomenon that has been occurred in several cases. However, presence of autoimmune disorders and IgG subclass deficiency in association with distinct HLA haplotypes could increase the risk of CVID induction in a genetically susceptible IgAD patient [63]. Thus, the screening of immunoglobulins is better implemented so that intravenous immunoglobulin (IVIG) therapy can be considered in case of disease progression towards CVID. Finally, the lack of an established screening method and the difficulty of diagnosis owing to the heterogeneity of the clinical presentation in PIgAD and SIgAD cloud a precise estimation of the patients in each category of IgA deficiency [64].

Moreover, new clinical manifestations might appear during patients’ follow-up, particularly in the presence of a positive family history [41], indicating that the age of patients and the course of the disease probably interfere with the reported frequency. In addition, periodic assessment in IgAD patients is warranted.

One of our limitations in the current study was the lack of information or incomplete data regarding the clinical manifestations and immunologic evaluations of IgAD cases in the published literature; information that could play a rather important role in categorizing different types of IgAD.

Overall, our study identified the infection as the most prevalent clinical presentation among IgAD patients, followed by allergic and autoimmune diseases. Due to the functional role of secretory IgA in maintaining the homeostasis of mucosal surfaces, these complications mainly involved respiratory and GI tracts. SIgAD patients tend to have altered intestinal and oropharyngeal microbiota that can be only partially compensated with IgM secretion [65, 66]. These microbial perturbations may play a role in developing or exacerbating immune dysregulations in the patients. The spectrum of clinical manifestations in PIgAD and SIgAD are mainly similar with a few minor discrepancies in allergic presentations. The coexistence of IgA and IgG subclass deficiency in patients may increase the susceptibility to infections. CVID and IgAD share clinical and immunological features; few IgAD patients progress toward CVID. Furthermore, new clinical manifestations could be detected in the course of the disease, implying the necessity of periodic assessment in these patients. However, the same size of the PIgAD and concurrent IgA and IgG subclass deficiency is small to conclude.

### Electronic supplementary material

Below is the link to the electronic supplementary material.

Supplementary Material 1

## Data Availability

All data generated or analyzed during this study are included in this published article.

## References

[1] Yel L (2010). Selective IgA deficiency. J Clin Immunol.

[2] Castrignano S, Carlsson B, Carneiro-Sampaio M, Söderström T, Hanson L (1993). IgA and IgG subclass deficiency in a poor population in a developing country. Scand J Immunol.

[3] Vo Ngoc DL, Krist L, van Overveld FJ, Rijkers GT (2017). The long and winding road to IgA deficiency: causes and consequences. Expert Rev Clin Immunol.

[4] Morawska I, Kurkowska S, Bębnowska D, Hrynkiewicz R, Becht R, Michalski A (2021). The epidemiology and clinical presentations of atopic diseases in selective IgA deficiency. J Clin Med.

[5] Abolhassani H, Aghamohammadi A, Hammarström L (2016). Monogenic mutations associated with IgA deficiency. Expert Rev Clin Immunol.

[6] Grosserichter-Wagener C, Franco‐Gallego A, Ahmadi F, Moncada‐Vélez M, Dalm VA, Rojas JL (2020). Defective formation of IgA memory B cells, Th1 and Th17 cells in symptomatic patients with selective IgA deficiency. Clin translational Immunol.

[7] Yazdani R, Latif A, Tabassomi F, Abolhassani H, Azizi G, Rezaei N (2015). Clinical phenotype classification for selective immunoglobulin A deficiency. Expert Rev Clin Immunol.

[8] Jorgensen G, Gardulf A, Sigurdsson M, Sigurdardottir ST, Thorsteinsdottir I, Gudmundsson S (2013). Clinical symptoms in adults with selective IgA deficiency: a case-control study. J Clin Immunol.

[9] Singh K, Chang C, Gershwin ME (2014). IgA deficiency and autoimmunity. Autoimmun rev.

[10] Abolhassani H, Gharib B, Shahinpour S, Masoom S, Havaei A, Mirminachi B (2015). Autoimmunity in patients with selective IgA deficiency. J Investigat Allergol Clin Immunol.

[11] Ludvigsson JF, Neovius M, Ye W, Hammarström L (2015). IgA deficiency and risk of cancer: a population-based matched cohort study. J Clin Immunol.

[12] Nabavizadeh SH, Karimi MH, Esmaeilzadeh H, Attarhoseini M, Askarisarvestani A (2021). The prevalence and clinical manifestations of IgA deficiency among blood donors at transfusion centers in Shiraz, Southern Iran. Am J Clin Experimental Immunol.

[13] Odineal DD, Gershwin ME (2020). The epidemiology and clinical manifestations of autoimmunity in selective IgA deficiency. Clin Rev Allergy Immunol.

[14] Wang N, Shen N, Vyse TJ, Anand V, Gunnarson I, Sturfelt G (2011). Selective IgA deficiency in autoimmune diseases. Mol Med.

[15] Petty R, Palmer N, Cassidy J, Tubergen D, Sullivan D (1979). The association of autoimmune diseases and anti-IgA antibodies in patients with selective IgA deficiency. Clin Exp Immunol.

[16] Mohammadinejad P, Pourhamdi S, Abolhassani H, Mirminachi B, Havaei A, Masoom S (2015). Primary antibody deficiency in a tertiary referral hospital: a 30-year experiment. J Investig Allergol Clin Immunol.

[17] Østergaard PA (1984). Alpha-1-antitrypsin levels and clinical symptoms in forty-eight children with selective IgA deficiency. Eur J Pediatrics.

[18] Kanoh T, Nishida O, Uchino H, Miyake T (1987). Immunoglobulin-producing cells in secretory immune system in patients with selective IgA deficiency. Gastroenterol Japonica.

[19] Zhang J, Kong W, Ni J, Guo Z, Yang X, Cao Q (2020). The epidemiology and clinical feature of selective immunoglobulin a deficiency of Zhejiang Province in China. J Clin Lab Anal.

[20] Mellemkjaer L, Hammarström L, Andersen V, Yuen J, Heilmann C, Barington T (2002). Cancer risk among patients with IgA deficiency or common variable immunodeficiency and their relatives: a combined danish and swedish study. Clin Experimental Immunol.

[21] Živković J, Lipej M, Banić I, Bulat Lokas S, Nogalo B, Lulić Jurjević R (2019). Respiratory and allergic disorders in children with severe and partial immunoglobulin a immunodeficiency. Scand J Immunol.

[22] Morell A, Muehlheim E, Schaad U, Skvaril F, Rossi E (1986). Susceptibility to infections in children with selective IgA-and IgA-IgG subclass deficiency. Eur J Pediatrics.

[23] Moschese V, Chini L, Graziani S, Sgrulletti M, Gallo V, Di Matteo G (2019). Follow-up and outcome of symptomatic partial or absolute IgA deficiency in children. Eur J Pediatrics.

[24] Nechvatalova J, Pikulova Z, Stikarovska D, Pesak S, Vlkova M, Litzman J (2012). B-lymphocyte subpopulations in patients with selective IgA deficiency. J Clin Immunol.

[25] Shkalim V, Monselize Y, Segal N, Zan-Bar I, Hoffer V, Garty BZ (2010). Selective IgA deficiency in children in Israel. J Clin Immunol.

[26] Janzi M, Kull I, Sjöberg R, Wan J, Melén E, Bayat N (2009). Selective IgA deficiency in early life: association to infections and allergic diseases during childhood. Clin Immunol.

[27] Plebani A, Monafo V, Ugazio A, Burgio GR (1986). Clinical heterogeneity and reversibility of selective immunoglobulin A deficiency in 80 children. The Lancet.

[28] Dambuza IM, Brown GD (2021). Managing the mycobiota with IgA. Nat Microbiol.

[29] Edwards E, Razvi S, Cunningham-Rundles C (2004). IgA deficiency: clinical correlates and responses to pneumococcal vaccine. Clin Immunol.

[30] Papadopoulou A, Mermiri D, Taousani S, Triga M, Nicolaidou P, Priftis KN (2005). Bronchial hyper-responsiveness in selective IgA deficiency. Pediatr Allergy Immunol.

[31] Erkoçoğlu M, Metin A, Kaya A, Özcan C, Akan A, Civelek E (2017). Allergic and autoimmune disorders in families with selective IgA deficiency. Turk J Med Sci.

[32] Wang W, Yao T, Zhang T, Quan M, Wang C, Wang C (2020). Selective immunoglobulin A deficiency (SIgAD) primarily leads to recurrent infections and autoimmune diseases: a retrospective study of chinese patients in the past 40 years. Genes & Diseases.

[33] Aytekin C, Tuygun N, Gokce S, Dogu F, Ikinciogullari A (2012). Selective IgA deficiency: clinical and laboratory features of 118 children in Turkey. J Clin Immunol.

[34] Yazdani R, Azizi G, Abolhassani H, Aghamohammadi A (2017). Selective IgA Deficiency: Epidemiology, Pathogenesis, clinical phenotype, diagnosis, prognosis and management. Scand J Immunol.

[35] Mella MA, Lavrinienko A, Akhi R, Hindström R, Nissinen AE, Wang C (2021). Compensatory IgM to the rescue: patients with selective IgA Deficiency have increased natural IgM antibodies to MAA-LDL and no changes in oral microbiota. Immunohorizons.

[36] Aghamohammadi A, Cheraghi T, Gharagozlou M, Movahedi M, Rezaei N, Yeganeh M (2009). IgA deficiency: correlation between clinical and immunological phenotypes. J Clin Immunol.

[37] Kaufman H, Hobbs J (1970). Immunoglobulin deficiencies in an atopic population. The Lancet.

[38] Gleeson M, Cripps A, Clancy R, Hensley M, Henry R, Wlodarczyk J (1995). The significance of transient mucosal IgA deficiency on the development of asthma and atopy in children. Adv Exp Med Biol.

[39] Van Asperen P, Gleeson M, Kemp A, Cripps A, Geraghty S, Mellis C (1985). The relationship between atopy and salivary IgA deficiency in infancy. Clin Exp Immunol.

[40] Magen E, Blum I, Waitman DA, Kahan N, Forer B (2021). Autoimmune inner ear disease among patients with selective IgA Deficiency. Audiol Neurotology.

[41] Cinicola BL, Pulvirenti F, Capponi M, Bonetti M, Brindisi G, Gori A (2022). Selective IgA Deficiency and Allergy: a fresh look to an Old Story. Medicina.

[42] Woof JM, Kerr MA (2006). The function of immunoglobulin A in immunity. J Pathology: J Pathological Soc Great Br Irel.

[43] Delavari S, Shariati S, Salami F, Rasouli S. Allergy in patients with selective IgA Deficiency. Immunol Genet J. 2020:54–63.

[44] Sarmiento E, Mora R, Rodríguez-Mahou M, Rodríguez-Molina J, Fernández-Cruz E, Carbone J (2005). Enfermedad autoinmune en inmunodeficiencias primarias de anticuerpos. Allergol Immunopathol.

[45] Jacob C, Pastorino AC, Fahl K, Carneiro-Sampaio M, Monteiro RC (2008). Autoimmunity in IgA deficiency: revisiting the role of IgA as a silent housekeeper. J Clin Immunol.

[46] Domínguez O, Giner M, Alsina L, Martín M, Lozano J, Plaza A, editors. Fenotipos clínicos asociados a la deficiencia selectiva de IgA: revisión de 330 casos y propuesta de un protocolo de seguimiento. Anales de Pediatría. Elsevier; 2012. 10.1016/j.anpedi.2011.11.006 22240193

[47] Arima H, Koirala S, Nema K, Nakano M, Ito H, Poudel KM (2022). High prevalence of rheumatoid arthritis and its risk factors among tibetan highlanders living in Tsarang, Mustang district of Nepal. J Physiol Anthropol.

[48] Caviglia GP, Garrone A, Bertolino C, Vanni R, Bretto E, Poshnjari A et al. Epidemiology of Inflammatory Bowel Diseases: a Population Study in a Healthcare District of North-West Italy. J Clin Med. 2023;12(2). 10.3390/jcm12020641 PMC9860659 36675570

[49] Singh P, Arora A, Strand TA, Leffler DA, Catassi C, Green PH (2018). Global prevalence of Celiac Disease: systematic review and Meta-analysis. Clin Gastroenterol Hepatol.

[50] Dominguez O, Giner M, Alsina L, Martin M, Lozano J, Plaza A, editors. Clinical phenotypes associated with selective IgA deficiency: a review of 330 cases and a proposed follow-up protocol. Anales de Pediatria (Barcelona, Spain: 2003); 2012. 10.1016/j.anpedi.2011.11.006 22240193

[51] Koskinen S (1996). Long-term follow-up of health in blood donors with primary selective IgA deficiency. J Clin Immunol.

[52] Azizi G, Tavakol M, Rafiemanesh H, Kiaee F, Yazdani R, Heydari A (2017). Autoimmunity in a cohort of 471 patients with primary antibody deficiencies. Expert Rev Clin Immunol.

[53] Ludvigsson JF, Neovius M, Hammarström L (2014). Association between IgA deficiency & other autoimmune conditions: a population-based matched cohort study. J Clin Immunol.

[54] Meini A, Pillan NM, Ugazio AG, Villanacci V, Monafo V, Plebani A (1996). Prevalence and diagnosis of celiac disease in IgA-deficient children. Ann Allergy Asthma Immunol.

[55] Mohammadi J, Ramanujam R, Jarefors S, Rezaei N, Aghamohammadi A, Gregersen PK (2010). IgA deficiency and the MHC: assessment of relative risk and microheterogeneity within the HLA A1 B8, DR3 (8.1) haplotype. J Clin Immunol.

[56] Kumar V, Jarzabek-Chorzelska M, Sulej J, Karnewska K, Farrell T, Jablonska S (2002). Celiac disease and immunoglobulin a deficiency: how effective are the serological methods of diagnosis?. Clin Vaccine Immunol.

[57] DLÁ ML, Spátola A, Gonzáles de Campos A (2021). Prevalence and characteristics of selective IgA deficiency in celiac patients. Revista de Gastroenterologia del Peru: Organo Oficial de la Sociedad de Gastroenterologia del Peru.

[58] Pallav K, Xu H, Leffler DA, Kabbani T, Kelly CP (2016). Immunoglobulin A deficiency in celiac disease in the United States. J Gastroenterol Hepatol.

[59] Swain S, Selmi C, Gershwin ME, Teuber SS (2019). The clinical implications of selective IgA deficiency. J translational Autoimmun.

[60] Chipps BE, Talamo RC, Winkelstein JA (1978). IgA, Deficiency, recurrent pneumonias, and Bronchiectasis: clinical conference in Pulmonary Disease from the Department of Pediatrics, the Johns Hopkins University School of Medicine. Chest.

[61] French M, Denis K, Dawkins R, Peter J (1995). Severity of infections in IgA deficiency: correlation with decreased serum antibodies to pneumococcal polysaccharides and decreased serum IgG2 and/or IgG4. Clin Experimental Immunol.

[62] Aghamohammadi A, Mohammadi J, Parvaneh N, Rezaei N, Moin M, Espanol T (2008). Progression of selective IgA deficiency to common variable immunodeficiency. Int Arch Allergy Immunol.

[63] Aghamohammadi A, Mohammadi J, Parvaneh N, Rezaei N, Moin M, Espanol T (2008). Progression of selective IgA deficiency to common variable immunodeficiency. Int Arch Allergy Immunol.

[64] Matsuda K, Arioka H, Kobayashi D (2020). Risk factors of partial IgA deficiency among low serum IgA patients: a retrospective observational study. Cent Eur J Immunol.

[65] Berbers R-M, Mohamed Hoesein FAA, Ellerbroek PM, van Montfrans JM, Dalm VASH, van Hagen PM et al. Low IgA Associated with Oropharyngeal Microbiota Changes and Lung Disease in primary antibody Deficiency. Front Immunol. 2020;11. 10.3389/fimmu.2020.01245 PMC7318304 32636843

[66] Catanzaro JR, Strauss JD, Bielecka A, Porto AF, Lobo FM, Urban A (2019). IgA-deficient humans exhibit gut microbiota dysbiosis despite secretion of compensatory IgM. Sci Rep.

[67] Savilahti E, Pelkonen P, Visakorpi J (1971). IgA deficiency in children: a clinical study with special reference to intestinal findings. Arch Dis Child.

[68] Lougaris V, Sorlini A, Monfredini C, Ingrasciotta G, Caravaggio A, Lorenzini T (2019). Clinical and laboratory features of 184 italian pediatric patients affected with selective IgA deficiency (SIgAD): a longitudinal single-center study. J Clin Immunol.

[69] Collins-Williams C, Chiu A, Varga E (1971). The relationship of a topic disease and immunoglobulin levels with special reference to selective IgA deficiency. Clin Experimental Allergy.

[70] Savilahti E, Pelkonen P, Verkasalo M, Koskimies S (1985). Selective deficiency of immunoglobulin A in children. Klinische Pädiatrie.

[71] Danon YL, Dinari G, Garty B-Z, Horodniceanu C, Nitzan M, Grunebaum M (1983). Cholelithiasis in children with immunoglobulin A deficiency: a new gastroenterologic syndrome. J Pediatr Gastroenterol Nutr.

[72] De Laat P, Weemaes C, Gonera R, Van Munster P, Bakkeren J, Stoelinga G (1991). Clinical manifestations in selective IgA deficiency in childhood. A follow-up report. Acta Paediatr Scand.

[73] Pituch-Noworolska A, Błaut-Szlósarczyk A, Zwonarz K (2013). Occurrence of autoantibodies for gastrointestinal autoimmune diseases in children with common variable immune deficiency and selected IgA deficiency. Gastroenterol Review/PrzeglÄ d Gastroenterologiczny.

[74] Jorgensen GH, Thorsteinsdottir I, Gudmundsson S, Hammarstrom L, Ludviksson BR (2009). Familial aggregation of IgAD and autoimmunity. Clin Immunol.

[75] López RV, Cid CM, García GR, Romero RG, Cilleruelo ML, Riechmann ER (2020). Influence of the 2012 european guidelines in diagnosis and follow-up of coeliac children with selective IgA deficiency. J Pediatr Gastroenterol Nutr.

[76] Klemola T, Savilahti E, Arato A, Ormälä T, Partanen J, Eland C (1995). Immunohistochemical findings in jejunal specimens from patients with IgA deficiency. Gut.

[77] Korponay-Szabó IR, Dahlbom I, Laurila K, Koskinen S, Woolley N, Partanen J (2003). Elevation of IgG antibodies against tissue transglutaminase as a diagnostic tool for coeliac disease in selective IgA deficiency. Gut.

[78] Gulez N, Gulez N, Karaca NE, Gulez N, Karaca NE, Aksu G (2009). Increased percentages of autoantibodies in immunoglobulin A-deficient children do not correlate with clinical manifestations. Autoimmunity.

[79] Beard LJ, Ferrante A, Oxelius V-A, Maxwell GM (1986). IgG subclass deficiency in children with IgA deficiency presenting with recurrent or severe respiratory infections. Pediatr Res.

