# Supplementary material for: Clinical manifestation for immunoglobulin A deficiency: a systematic review and meta-analysis
Source: Allergy Asthma Clin Immunol. 2023 Aug 28;19:75. doi: 10.1186/s13223-023-00826-y (PMC10463351; doi:10.1186/s13223-023-00826-y)
Supplement: Supplementary file 1 — Supplementary Material 1 [file 13223_2023_826_MOESM1_ESM.docx]

**Suplimentary material**

**Figure S1.** The forest plot and pooled prevalence of the bronchiectasis in SIgAD.

**Table S1.** Main characteristics of the included studies on the prevalence of manifestation in SIgAD patients.

| **First author (year of publication)** | **Country** | **Study design** | **Recruitment setting and methods** | **Final sample size (male and female)** | **Age characteristics for the total sample, years** | **Infection n (%)** | **Autoimmunity n (%)** | **Allergic diseases**  **n (%)** | **Malignancy n (%)** |
| --- | --- | --- | --- | --- | --- | --- | --- | --- | --- |
| E Magen (2021) | Israel | Case-Control | Leumit Healthcare Services | 347 (192/155) | Mean age = 24.18 | -* | 105 | 161 | - |
| RV López  (2020) | Spain | Cohort | Hospital Universitario  Clinico San Carlos | 86 (44/42) | Mean age = 4.3 | - | - | - | - |
| Zhang J.  (2020) | China | Cross-sectional | 9 hospitals in Zhejiang  Province | 109 (46/63) | Mean age = 43.22 | 63 (57.79) | 35 (32.11) | 6 (5.50) | 4 (3.66) |
| Wang W.  (2020) | China | Cross-sectional | Peking Union Medical  College Hospital | 43 (19/24) | Mean age = 13.0 | 29 (67.44) | 13 (30.23) | 6 (13.95) | 3 (6.97) |
| Lougaris V.  (2019) | Italy | Cross-sectional | Department of Clinical and Experimental Sciences, University of  Brescia | 184 (104/80) | Mean age = 8.9 | - | - | - | - |
| Živković J.  (2019) | Croatia | Cross-sectional | Paediatric Clinic of Pulmonology, Allergology and  Immunology at Srebrnjak Children's Hospital (SCH) | 45 (24/21) | - | - | - | - | - |
| Moschese V.  (2018) | Italy | Cohort | the Pediatric Immunopathology and Allergology Unit of  Policlinico of Tor Vergata in Rome, at the Department of  Pediatrics of Malpighi Hospital in Bologna, and at the  Pediatric Immunology Center of Federico II University  in Naples | 53 (31/22) | Mean age = 7.0 | 44 (83.0) | 4 (7.54) | 19 (35.84) | 0 |
| Erkoçoğlu M.  (2017) | Turkey | Cross-sectional | Ankara Children’s  Hematology Oncology Education and Research Hospital | 81 | Mean age = 10.4 | 60 (74.07) | 14 (17.28) | 37 (45.67) | - |
| Mohammadinejad P.  (2015) | Iran | Cross-sectional | Tehran  University of Medical Sciences | 63 (46/17) | Mean age = 11 (5-36) | 62 (98.41) | 14  (22.22) | 43  (68.25) | 0 |
| Ludvigsson J.F.  (2015) | Sweden | Cohort | six university  hospitals in Sweden | 2100 (901/1199) | - | - | - | - | - |
| Bienvenu F.  (2014) | France | Original | Paediatric Hospital Gastroenterology  Hepatology Nutrition Department | 45 (27/18) | Mean age = 8.2 | - | - | - | - |
| Pituch-Noworolska A.  (2013) | Poland | Original | Department of Clinical Immunology, Polish-American Institute of Pediatrics, Jagiellonian University Medical College | 63 (38/25) | Mean age = 7.92 | - | - | - | - |
| G. H. Jorgensen  (2013) | Iceland | Case-Control | Department of Medicine, University of Iceland, Reykjavík, Iceland | 32 (19/13) | Mean age = 48 | - | - | - | - |
| Aytekin C.  (2012) | Turkey | Cross-sectional | Dr. Sami Ulus Maternity and Children’s Health and Diseases Training and Research Hospital | 118 (63/55) | Mean age = 7 | 99 (83.9) | 20 (17) | 51 (43.2) | - |
| Nechvatalova J.  (2012) | Czech Republic | Original | Department of Clinical Immunology and Allergology, St. Anne’s University Hospital | 80 (27/53) | Mean age = 33 | - | 13 (16.25) | 27 (33.75) | - |
| Shkalim V.  (2010) | Israel | Cohort | Department of Pediatrics B, Schneider Children’s Medical Center of Israel | 63 (42/21) | - | 25 (39.68) | 13 (20.63) | 20 (31.74) | 3 |
| Jorgensen G.H.  (2009) | Iceland | Cohort | Department of Medicine, University of Iceland, Reykjaví Iceland | 43 | - | - | 8 (18.60) | - | - |
| Gulez N.  (2009) | Turkey | Original | Department of Pediatrics, Faculty of Medicine, Ege University | 9** (5/4) | Mean age = 8.5 | - | - | 1 (11.11) | - |
| Cristina M. A. Jacob  (2008) | Brazil | Cross-sectional | Instituto da Criança, Hospital das Clinicas da Faculdade de Medicina,  Universidade de São Paulo | 126 | - | 80 (63.5) | 24 (19.0) | 61 (48.0) | - |
| Papadopoulou A.  (2005) | Greece | Cohort | Department of Allergology-Pulmonology, Penteli  Children's Hospital, P. Penteli | 20 (12/8) | Mean age = 12.6 | 9 (45.0) | - | - | - |
| Edwards E.  (2004) | USA | Cross-sectional | Mount Sinai Medical Center for consultation | 127 (59/68) | Mean age = 27 | 63 (49.6) | 34 (28.0) | 16 (12.59) | - |
| Korponay-Szabo I.R.  (2003) | Hungary | Original | the Heim Pa´l Children’s Hospital, Budapest | 325 | - | - | - | - | - |
| Majkowska-Skrobek G.  (2003) | Poland | Original | Department of General Microbiology, Institute of Genetics and Microbiology, University of Wroclaw | 21 (11/10) | Mean age = 8.97 | - | - | - | - |
| Mellemkjaer L.  (2002) | Denmark, Sweden | Cohort | Institute of Cancer Epidemiology, Danish Cancer Society, Copenhagen | 386 (172/214) | - | - | - | - | 28 (7.25) |
| Koskinen S.  (1996) | Finland | Cohort | Finnish Red Cross Blood Transfusion Service | 159 | - | - | 36 (22.64) | - | - |
| Klemola T.  (1995) | Finland | Original | The University of Helsinki,  Helsinki, Finland | 25 (15/10) | Mean age = 9.8 | - | - | - | - |
| De Laat P.C.  (1991) | Netherland | Cross-sectional | the Department of Paediatrics, University Hospital Nijmegen | 40 (24/16) | - | - | - | 7 (17.5) | - |
| Kanoh T.  (1987) | Japan | Original | Department of Internal Medicine, Kyoto University | 10 (3/7) | Mean age = 25.6 | - | 4 (40) | - | 2 (20) |
| Plebani A.  (1986-1987) | Italy | Cross-sectional | Department of Pediatrics, University of Pavia and University of  Brescia, Italy | 40 (29/11) | Mean age = 5.8 | 24 (60) | - | 13 (32.5) | - |
| A Morell  (1986) | Switzerland | Cross-sectional | the University Children's Hospital in Berne | 26 | - | 23 (88.46) | 6 (23.07) | 2 (7.69) | - |
| Savilahti E.  (1985) | Finland | Cross-sectional | Children's Hospital, University of Helsinki | 74 | - | - | 29 (39.18) | - | - |
| Østergaard P.A.  (1984) | Demark | Original | Department of Pediatrics, Aalborg Hospital North | 48 | Mean age = 8.17 | - | 2 (4.16) | 19 (39.58) | - |
| Danon Y.L.  (1983) | Israel | Case series | Tel-Aviv University, Sackler School of Medicine | 13 (8/5) | Mean age = 6.5 | - | - | - | - |
| Petty R.E.  (1979) | Canada | Cross-sectional | Department Of Paediatrics and the Rheumatic Diseases Unit, University of Manitoba | 83 | - | 10 (12.04) | - | - | - |
| Savilahti E.  (1971) | Finland | Cross-sectional | Children's Hospital, The University of Helsinki | 26 (7/9) | - | - | 9 (34.61) | - | - |
| Collins-Williams C.  (1971) | Canada | Cross-sectional | Department of Paediatrics, Division of Paediatric Allergy,  The Hospital for Sick Children, Toronto | 27 (12/15) | - | - | - | 9 (33.33) | - |
| *The dash sign does not necessarily mean the absence of information about the variable, but in some articles, the total number of variables was not mentioned and also could not be calculated and concluded. Therefore, despite the existence of the information, it is not possible to reach the overall prevalence of those clinical manifestations.  ** In this study, 9 patients are SIgAD and 51 patients are PIgAD. | | | | | | | | | |

**Table S2.** The pooled prevalence of other complications in selective IgA deficiency patients

|  | **Ref** | **N*, sample size** | **ES% (95%CI); I2%** |
| --- | --- | --- | --- |
| Interstitial lung disease (ILD) |  | 0 |  |
| Splenomegaly | (16) | 1, 63 | 0.0 (0.0 to 5.75); NA |
| Hepatomegaly | (16) | 1, 63 | 0.0 (0.0 to 5.75); NA |
| Lymphadenopathy | (16, 72) | 2, 103 | 3.79 (0.65 to 8.72); NA |
| Failure to thrive | (16, 25) | 2, 126 | 1.59 (0.0 to 4.90); NA |
| Enteropathy | (16, 19, 33, 70) | 4, 364 | 12.73 (3.42 to 26.39); 90.83 |
| Chronic diarrhea | (45, 71, 72, 75) | 4, 265 | 21.08 (4.47 to 44.60); 92.80 |
| Nodular lymphoid hyperplasia |  | 0 |  |
| Chronic atrophic gastritis | (8, 19, 25, 33, 73) | 5, 385 | 5.13 (1.71 to 9.97); 65.41 |
| Lymphoproliferative | (16) | 1, 63 | 9.52 (4.44 to 19.26); NA |

*. Number of studies in Meta-analysis

**Table S3.** The pooled prevalence of reported complications in the Partial IgAD patients.

|  | **Ref** | **N*, sample size** | **ES% (95%CI); I2%** |
| --- | --- | --- | --- |
| **Autoimmunity/inflammatory** | (22, 23) | 2, 60 | 12.87 (4.83 to 23.34); NA |
| Autoimmune thyroiditis | (22, 23) | 2, 60 | 2.09 (0.0 to 8.68); NA |
| IDDM | (22, 23) | 2, 60 | 0.0 (0.0 to 2.07); NA |
| Celiac disease | (22, 23) | 2, 60 | 6.66 (0.97 to 15.40); NA |
| Rheumatoid arthritis | (22, 23) | 2, 60 | 2.09 (0.0 to 8.68); NA |
| **Infection** | (22, 23, 27) | 3, 100 | 66.36 (34.34 to 92.18); 88.44 |
| Respiratory tract infection | (22, 23, 27) | 3, 100 | 59.10 (17.56 to 94.44); 93.79 |
| Pneumonia | (27) | 1, 40 | 10.0 (3.96 to 23.05); NA |
| Gastrointestinal infection | (22, 23, 27) | 3, 100 | 13.92 (5.48 to 24.84); 36.46 |
| Viral infection | (23) | 1, 50 | 4.0 (1.10 to 13.46); NA |
| Parasitic infection | (23) | 1, 50 | 8.0 (3.15 to 18.84); NA |
| Fungal infection | (23) | 1, 50 | 4.0 (1.10 to 13.46); NA |
| **Malignancy** | (23) | 1, 50 | 0.0 (0.0 to 7.13); NA |
| **Allergic diseases** | (22, 23, 27, 78) | 4, 151 | 24.41 (12.50 to 38.50); 66.94 |
| Asthma | (21, 23, 27) | 3, 140 | 27.14 (3.84 to 60.29); 93.86 |
| Allergic rhinitis | (21, 23) | 2, 100 | 46.55 (36.76 to 56.48); NA |
| Allergic conjunctivitis | (23) | 1, 50 | 6.0 (2.06 to 16.22); NA |
| Eczema | (21, 23, 27) | 3, 140 | 9.96 (2.23 to 21.60); 71.91 |

*. Number of studies in Meta-analysis

**Table S4.** The pooled prevalence of reported complications in IgA deficient patients with IgG subclass deficiency

|  | **Ref** | **N*, sample size** | **ES% (95%CI); I2%** |
| --- | --- | --- | --- |
| **Autoimmunity/inflammatory** | (2) | 1, 17 | 17.65 (6.19 to 41.03); NA |
| Rheumatoid arthritis | (2) | 1, 17 | 5.88 (1.05 to 26.98); NA |
| SLE | (2) | 1, 17 | 5.88 (1.05 to 26.98); NA |
| **Infection** | (22, 79) | 2, 26 | 100.0 (95.26 to 100.0); NA |
| Respiratory tract infection | (2, 22, 79) | 3, 43 | 87.76 (31.08 to 100.0); 91.56 |
| Pneumonia | (2, 22) | 2, 21 | 26.81 (8.03 to 49.98); NA |
| Otitis | (22) | 1, 4 | 50.0 (15.0 to 85.0); NA |
| Bacterial infection | (79) | 1, 22 | 18.18 (7.31 to 38.52); NA |
| Viral infection | (79) | 1, 22 | 13.64 (4.75 to 33.33); NA |
| **Bronchiectasis** | (79) | 1, 22 | 4.55 (0.81 to 21.80); NA |
| **Chronic diarrhea** | (2) | 1, 17 | 11.76 (3.29 to 34.34); NA |
| **Malignancy** | (79) | 1, 22 | 0.0 (0.0 to 14.87); NA |
| **Allergic diseases** | (2) | 1, 17 | 17.6 (6.2 to 41.0); NA |
| Asthma | (79) | 1, 22 | 13.6 (4.7 to 33.3); NA |

*. Number of studies in Meta-analysis
